# Supplementary material for: Differential regulation of β-catenin-mediated transcription via N- and C-terminal co-factors governs identity of murine intestinal epithelial stem cells
Source: Nat Commun. 2021 Mar 1;12:1368. doi: 10.1038/s41467-021-21591-9 (PMC7921392; doi:10.1038/s41467-021-21591-9)
Supplement: Supplementary file 1 — Supplementary Information [file 41467_2021_21591_MOESM1_ESM.pdf]

Supplementary Information

Supplementary Fig. 1

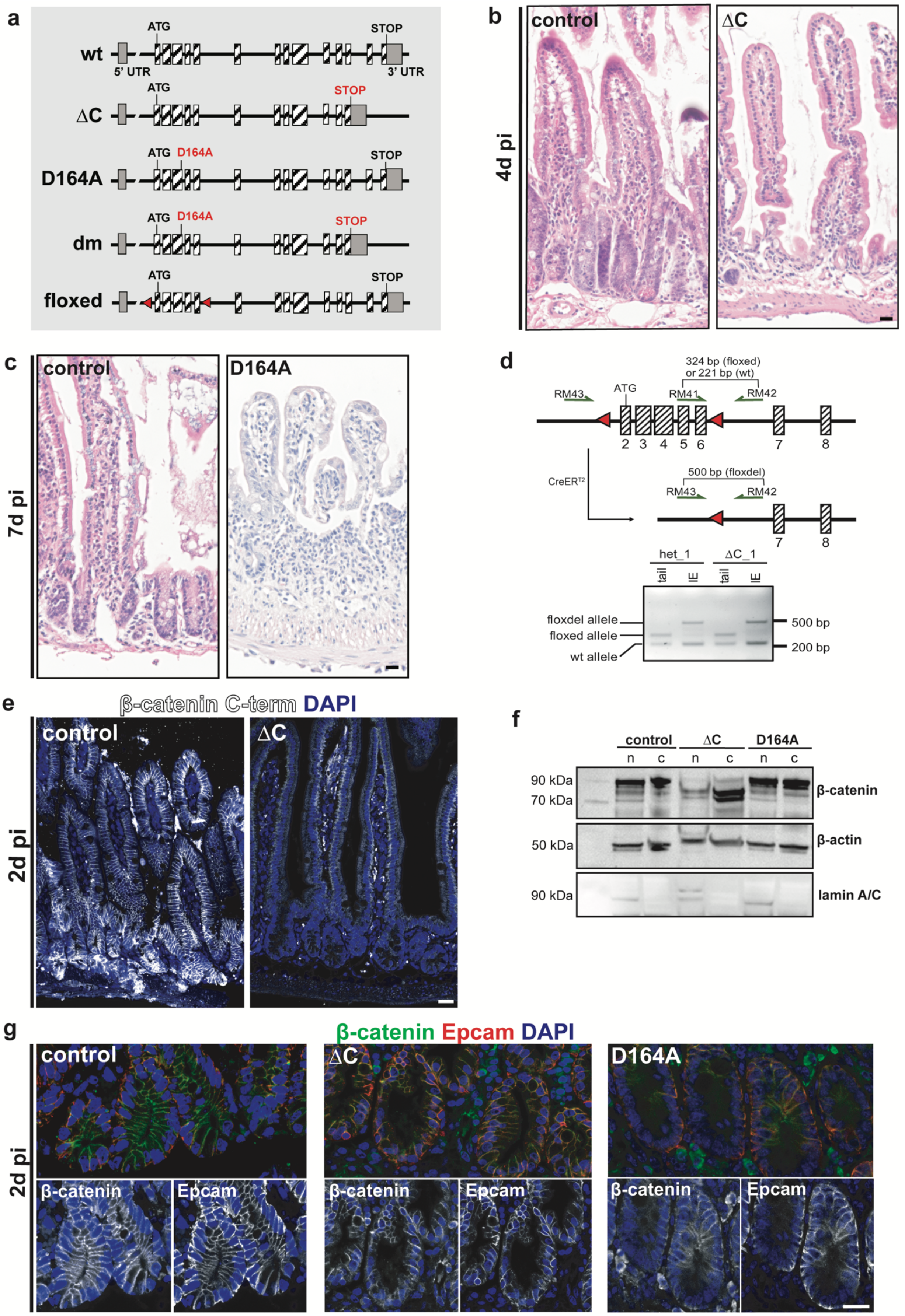

Supplementary Fig. 1. Intestinal-epithelial specific recombination of floxed β-catenin allele leads to sole presence of mutant β-catenin in intestinal crypts.

a) Scheme of the wt, mutant (ΔC, D164A and dm) and conditional (floxed) *Ctnnb1* knock-in alleles used in this study.

b) ΔC animals (*villin-CreER^{T2};Ctnnb1$^{\Delta C/flox}$*) suffer from crypt atrophy and reach humane endpoint 4d pi. Control animals (*villin-CreER^{T2};Ctnnb1$^{wt/flox}$*) show no overt phenotype. Scale bar, 20 μM. Representative images of 3 biological replicates.

c) D164A (*villin-CreER^{T2};Ctnnb1$^{D164A/flox}$*) suffer from crypt atrophy and villus shortening and reach humane endpoint 7d pi. Thickening of the mesenchyme indicates immune infiltration and severe colitis. Scale bar, 20 μM. Representative images of 3 biological replicates.

d) Map of the β-catenin locus, modified from[1] depicting how the recombination of conditional allele was confirmed. Red triangles indicate the loxP sequences flanking exons 2 (which contains the ATG) to 6. Prior to recombination, primers RM41 and RM42 generate a 221 bp band for the wt allele and a 324 bp band for the floxed allele, respectively. Upon Cre induction, primers RM42 and RM43 generate a 500 bp band. Representative PCR showing successful and tissue-specific recombination in DNA obtained from the intestinal epithelium (IE) but not from the tail.

e) wt β-catenin protein is depleted 2d pi in the crypts of ΔC animals. C-terminally truncated β-catenin is not recognized by the antibody used for this staining. Timepoint: 2d pi. Scale bar, 20 μM. Representative image of 2 biological replicates.

f) Western blot shows similar levels of control and mutant β-catenin in nuclear and cytosolic fractions obtained from control (*villin-CreER^{T2};Ctnnb1$^{wt/flox}$*), ΔC (*villin-CreER^{T2};Ctnnb1$^{\Delta C/flox}$*) and D164A (*villin-CreER^{T2};Ctnnb1$^{D164A/flox}$*) duodenal crypts. Timepoint: 2d pi. The antibody used here binds β-catenin's N-terminus and thus recognizes C-terminally truncated mutant β-catenin, which exhibits a 12 kDa shift in molecular weight. Of note, no wt β-catenin is found in ΔC lysates. β-actin serves as loading control. Lamin A/C is enriched in nuclear fractions. Source data are provided as a Source Data file. Representative blot of 3 biological replicates.

g) wt β-catenin, β-catenin-ΔC and β-catenin-D164A co-localize with the epithelial cell adhesion molecule Epcam at the cell membrane. Timepoint: 2d pi. Scale bar: 20 μM. Representative images of 3 biological replicates.

Supplementary Fig. 2.

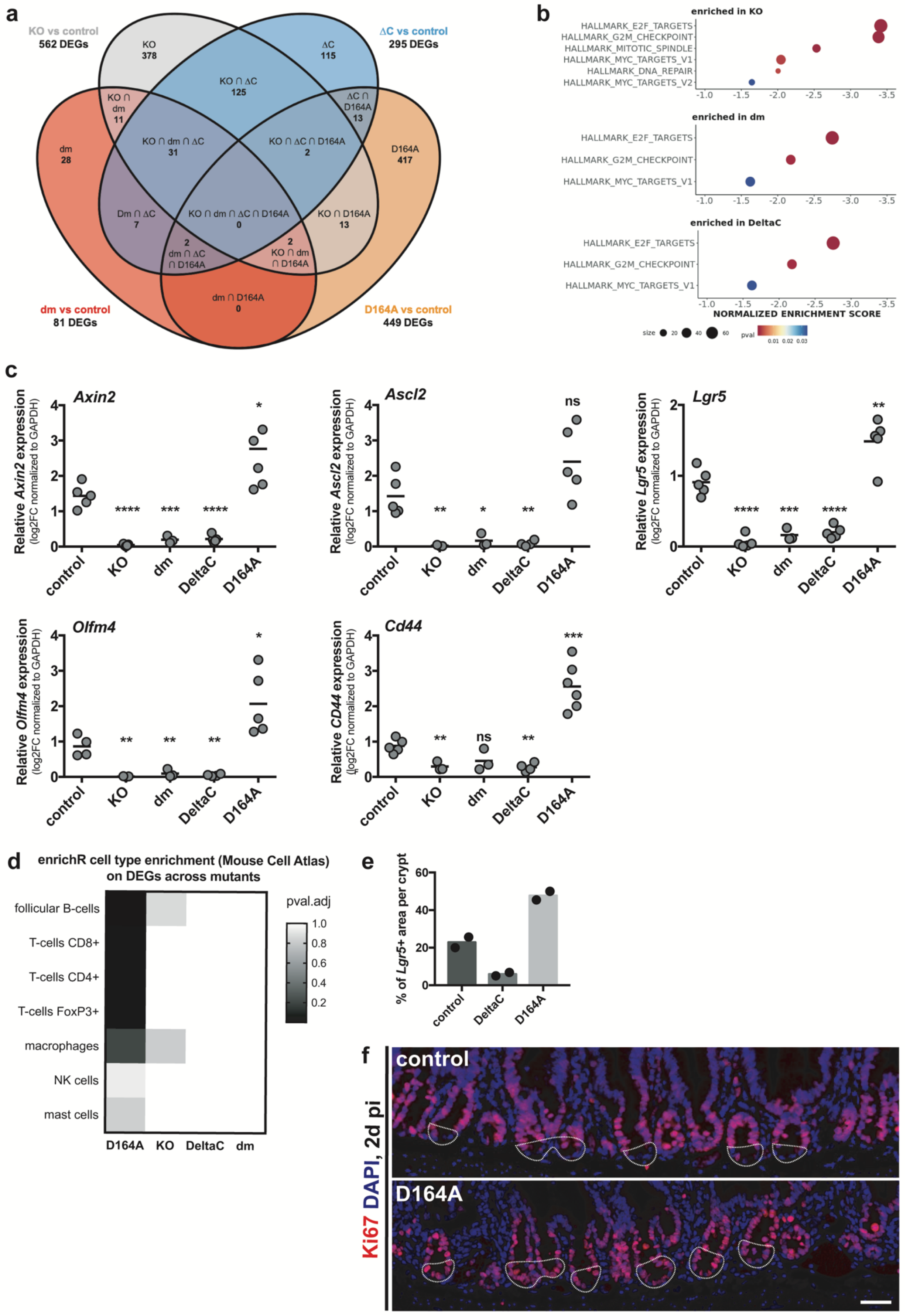

Supplementary Fig. 2. Distinct effects of N- vs. C-terminal β-catenin transcriptional outputs on intestinal homeostasis.

a) Venn diagram of intersecting differentially expressed genes (DEGs) (logFC > |2|, $p < 0.01$, as calculated by edgeR[2]'s exact test) in ΔC (*villin-CreER^{T2};Ctnnb1$^{\Delta C/flox}$*, n=2), dm (*villin-CreER^{T2};Ctnnb1$^{dm/flox}$*, n=3), KO (*villin-CreER^{T2};Ctnnb1$^{flox/flox}$*, n=2), D164A (*villin-CreER^{T2};Ctnnb1$^{D164A/flox}$*, n=3), with respect to control (*villin-CreER^{T2};Ctnnb1$^{wt/flox}$*, n=3). Timepoint: 2d pi.

b) Gene set enrichment analysis (GSEA) on DEGs (logFC > |2|, $p < 0.01$, as calculated by two-sided hypergeometric test) of KO, dm and ΔC animals. Annotated gene sets obtained from the Hallmarks collection of the Molecular Signatures Database (MSigDB).

c) qRT-PCR of Wnt target genes in D164A (n=6), ΔC (n=4), dm (n=3), KO (n=3) and control (n=5) crypts. Timepoint: 2d pi. Expression levels normalized to *GAPDH*. **** $p < 0.0001$, *** $p < 0.001$, ** $p < 0.01$, * $p < 0.05$, as calculated by two-sided, unpaired Student's T-test (KO). Horizontal line indicates mean expression.

d) Cell type enrichment analysis in DEGs across mutants performed on the web-based tool EnrichR[3,4].

e) Percentage of *Lgr5*+ area per crypt in control and D164A animals, n=2. Barplot indicates mean area.

f) Ki67 (red) and DAPI (blue) immunofluorescence in control and D164A small intestinal sections. Dashed lines indicate increased Ki67+ cells at the crypt base of D164A mutants. Scale bar, 20 μM.

Supplementary Fig 3.

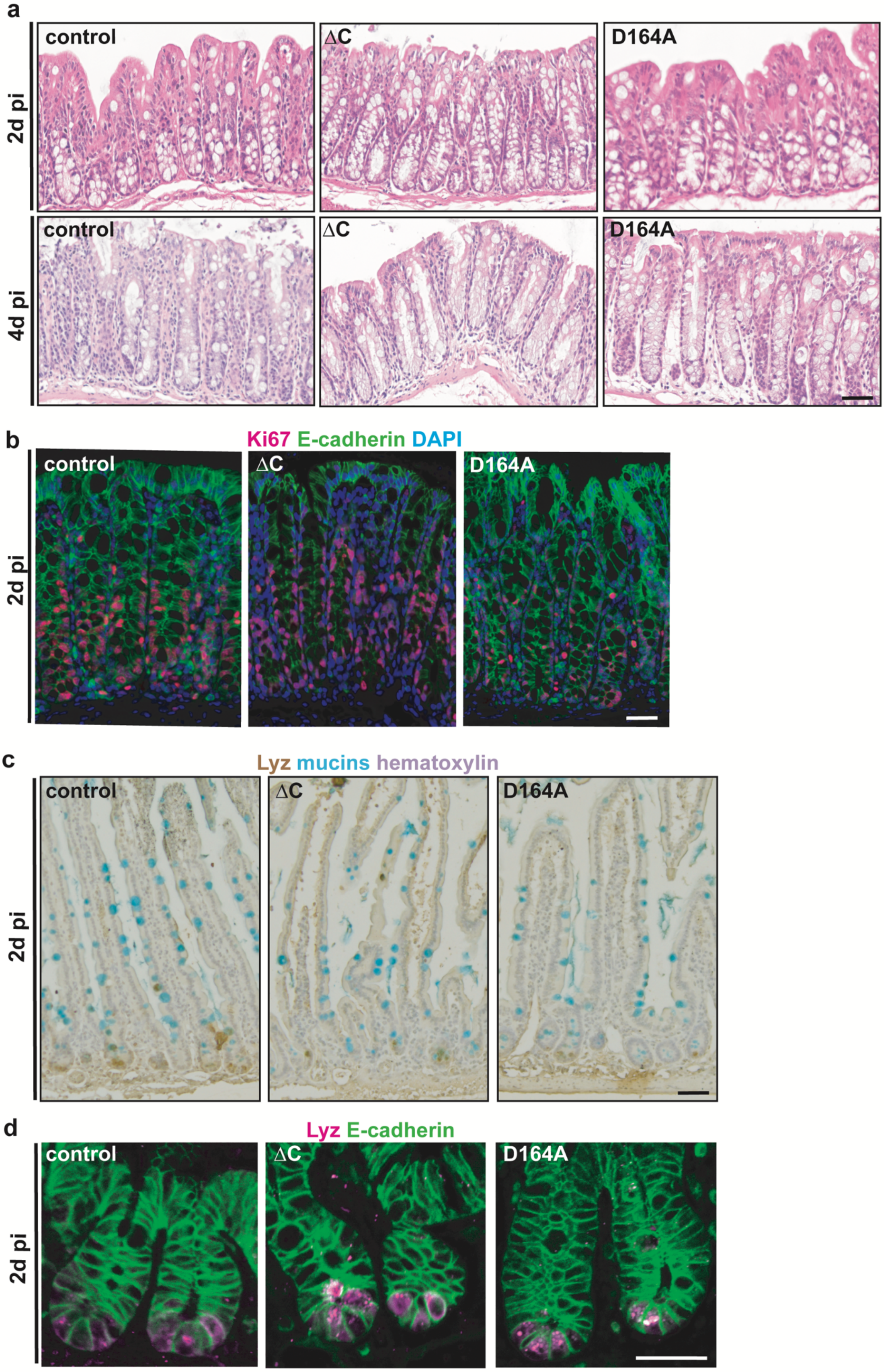

Supplementary Fig 3. The effect of β-catenin mutations 2d pi is only apparent in small intestinal IESCs.

a) H&E staining of control (*villin-CreER^{T2};Ctnnb1$^{wt/flox}$*), ΔC (*villin-CreER^{T2};Ctnnb1$^{\Delta C/flox}$*) and D164A (*villin-CreER^{T2};Ctnnb1$^{D164A/flox}$*) colon shows no overt epithelial morphology at timepoints 2d and 4d pi. Representative images of 2 biological replicates. Scale bar, 20 μM.

b) Ki67 staining does not indicate any proliferative change in the colonic epithelium of β-catenin mutants. Timepoint: 2d pi. DAPI (nuclei) or E-cadherin (cell shape) for counterstain. Scale bar, 20 μM. Representative images of 2 biological replicates.

c) Paneth cells and goblet cells in the small intestinal epithelium of control, ΔC and D164A animals visualized by Lyz-HRP and alcian blue (mucins), respectively. Timepoint: 2d pi. Hematoxylin as counterstain. Scale bar, 20 μM. Representative images of 3 biological replicates.

d) Lysozyme (Lyz) immunofluorescence in control, ΔC and D164A crypts. Timepoint: 2d pi. E-cadherin (cell shape) for counterstain. Scale bar, 20 μM. Representative images of 3 biological replicates.

Supplementary Fig 4.

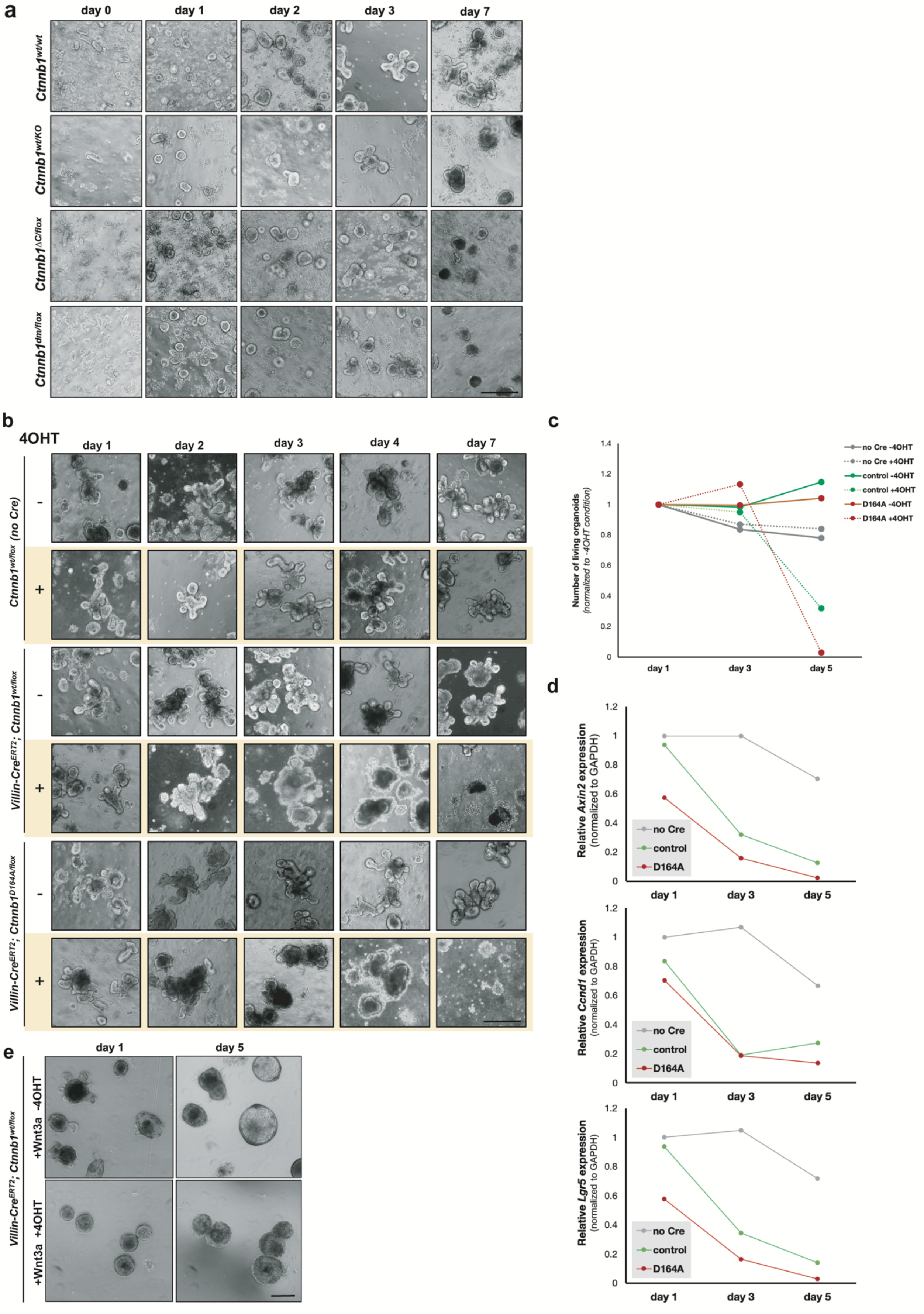

Supplementary Fig 4. β-catenin is haploinsufficient *in vitro*.

a) Duodenal organoids derived from *Ctnnb1*$^{\Delta C/flox}$ and *Ctnnb1*$^{dm/flox}$, as well as from constitutively hemizygous *Ctnnb1*$^{KO/wt}$ animals grow slower than wt controls and can not be propagated *in vitro*. Scale bar, 20 µM. Representative images of 2 biological replicates.

b) *villin-CreER*^{T2}*;Ctnnb1*$^{wt/flox}$ and *villin-CreER*^{T2}*;Ctnnb1*$^{D164A/flox}$ organoids treated with 4-hydroxytamoxifen (4OHT) *in vitro* die 7 days after induction of recombination. Duodenal organoids lacking the *villin-CreER*^{T2} allele are not affected by 4OHT. Days after addition of 4OHT indicated at the top. Scale bar, 20 µM. Representative images of 2 biological replicates.

c) Quantification of living organoids upon 4OHT addition. Abbreviations: control = *villin-CreER*^{T2}*;Ctnnb1*$^{wt/flox}$, no Cre = *Ctnnb1*$^{wt/flox}$, D164A = *villin-CreER*^{T2}*;Ctnnb1*$^{D164A/flox}$.

d) qRT-PCR of *Axin2* (Wnt target gene), *Ccnd1* (proliferative marker), and *Lgr5* (IESCs marker) genes in D164A and control organoids upon addition of 4OHT. Expression levels normalized to *GAPDH*.

e) *villin-CreER*^{T2}*;Ctnnb1*$^{wt/flox}$ organoids grown in medium in Wnt3a-conditioned medium die 5 day after 4OHT addition. Scale bar, 20 µM. Representative images of 2 biological replicates.

Supplementary Fig. 5.

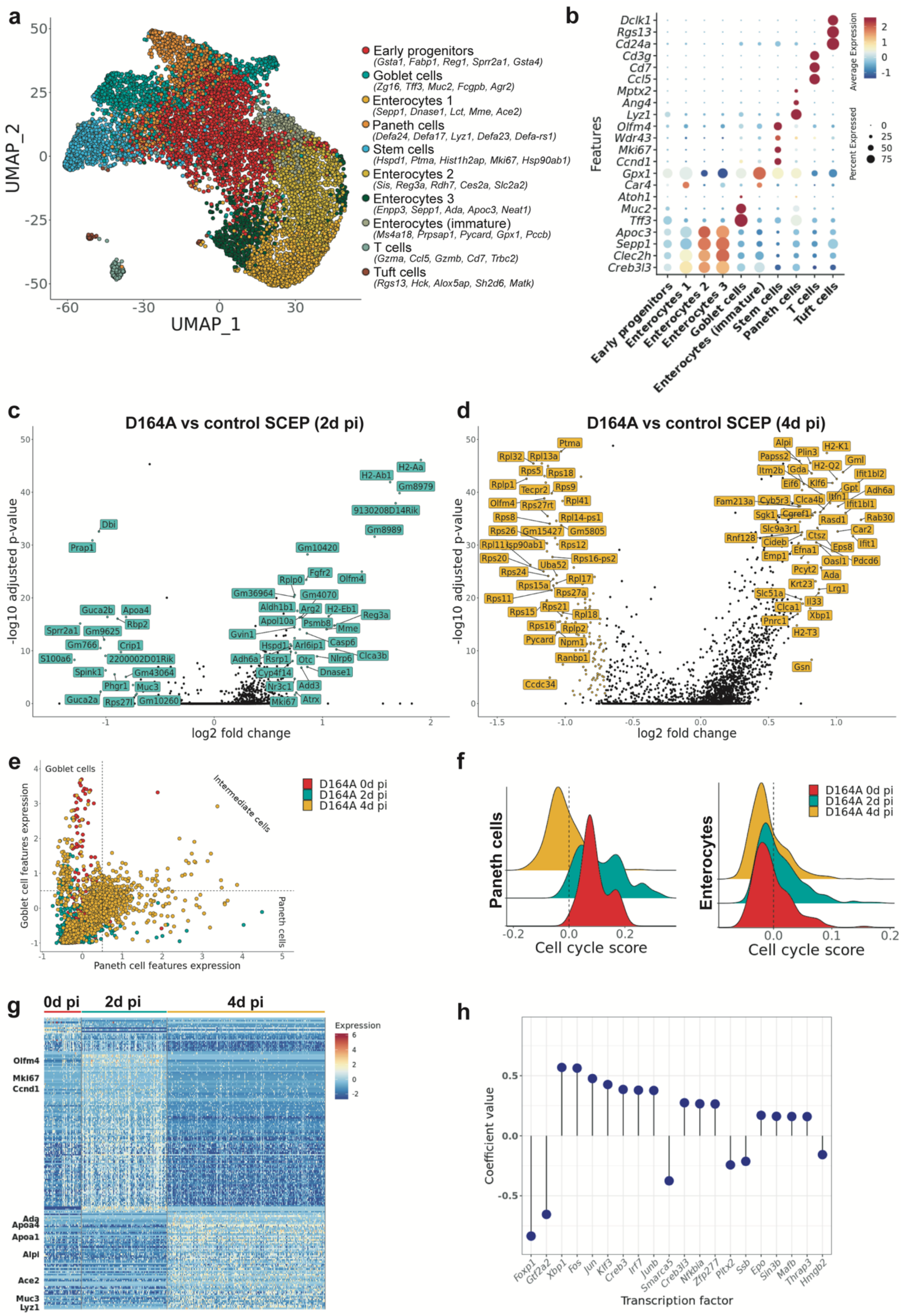

Supplementary Fig. 5. Longitudinal scRNASeq of control and N-terminal mutant (D164A) crypts.

a) UMAP embedding of joint graph obtained from single cells sequenced from control (*villin-CreER^{T2};Ctnnb1$^{wt/flox}$*, n=3) and D164A (*villin-CreER^{T2};Ctnnb1$^{D164A/flox}$*, n=3) crypts isolated 0, 2 and 4d pi. Leiden clustering results in 10 clusters corresponding to the main cell populations in the intestinal crypt. 5 top marker genes of each cluster indicated below each cluster name.

b) Marker gene expression across clusters.

c) Volcano plot of differentially expressed genes between D164A and control SCEP cells 2d pi, as calculated by Seurat's FindMarker function (Wilcoxon Rank Sum test).

d) Volcano plot of differentially expressed genes between D164A and control SCEP cells 4d, as calculated by Seurat's FindMarker function (Wilcoxon Rank Sum test).

e) Intermediate cells show co-expression of goblet (y-axis) and Paneth cell (x-axis) features and are uniquely found 4d pi.

f) Distribution of cell cycle score (average expression of cycling genes) in Paneth cells and enterocytes over time.

g) Heatmap of the top 200 differentially regulated genes in normalized D164A stem cells and early progenitors (SCEP) cells across timepoints. IESC and proliferation markers and upregulated 2d pi, while expression of differentiation markers increases 4d pi.

h) Supervised pseudotime ordering coefficients of mouse transcription factors in decreasing absolute value.

Supplementary Fig. 6.

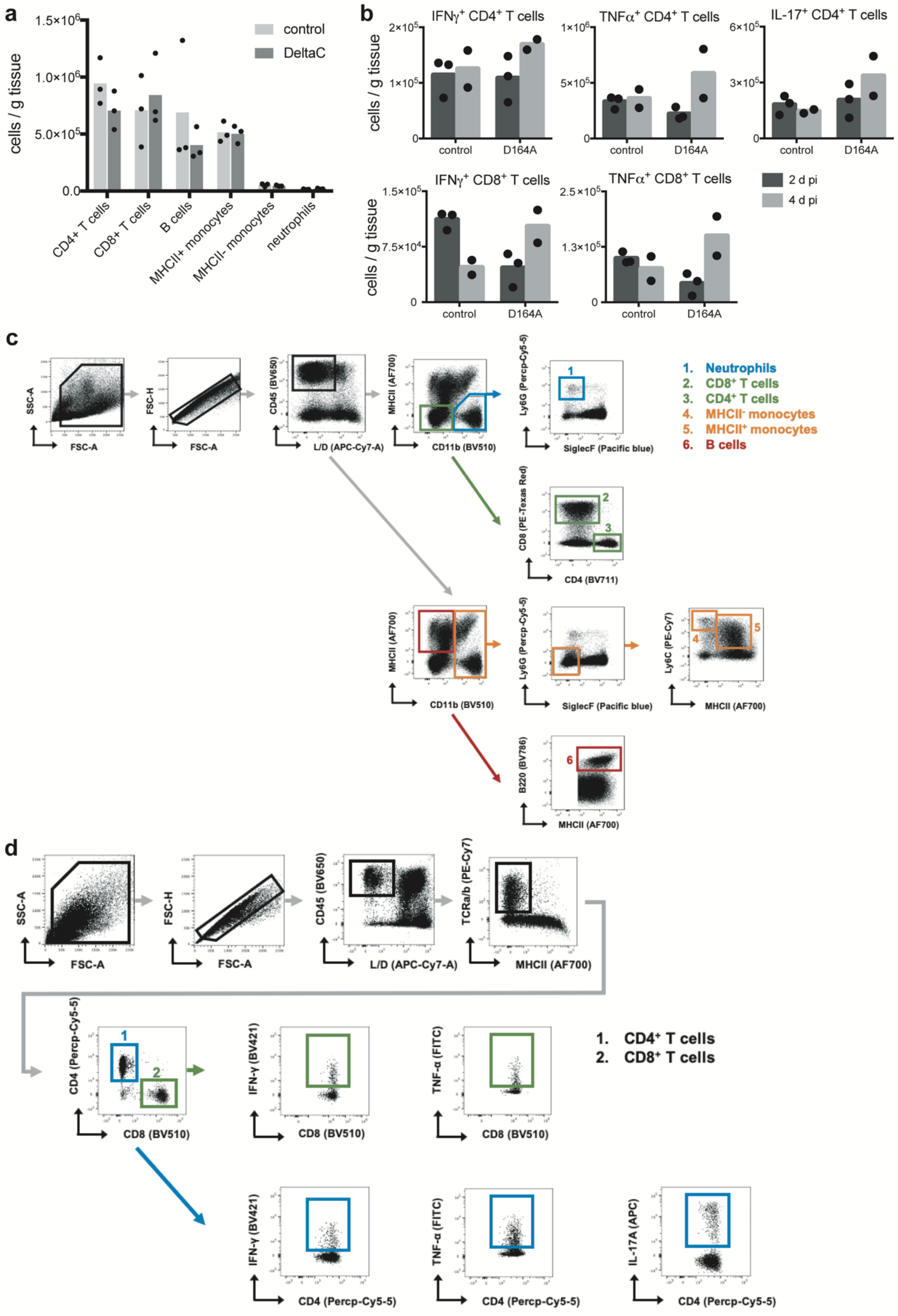

Supplementary Fig. 6. Leukocyte profiling in small intestines of mutant animals.

a) Counts per mg of tissue of $CD4^+$ and $CD8^+$ T-cell, B-cells, $MHCII^+$ and $MHCII^-$ monocytes, and neutrophils in ΔC (*villin-CreER^{T2};Ctnnb1$^{\Delta C/flox}$*, n=3) and control small intestine (*villin-CreER^{T2};Ctnnb1$^{wt/flox}$*, n=3). Timepoint: 2d pi. The control values used here are the same as in main figure 5. Barplots show mean counts.

b) Counts per mg of tissue of IFN-γ^+, TNF-α^+ and IL-17^+ $CD4^+$ and $CD8^+$ T-cells isolated from the small intestine of D164A (*villin-CreER^{T2};Ctnnb1$^{D164A/flox}$*) and control (*villin-CreER^{T2};Ctnnb1$^{wt/flox}$*) animals. Timepoints: 2d (n=3) and 4d pi (n=2). Barplots show mean counts.

c) Gating strategy for leukocyte profiling shown in Fig. 6e,f.

d) Gating strategy for T-cell re-stimulation shown in Supplementary Fig. 6a.

Supplementary Fig. 7.

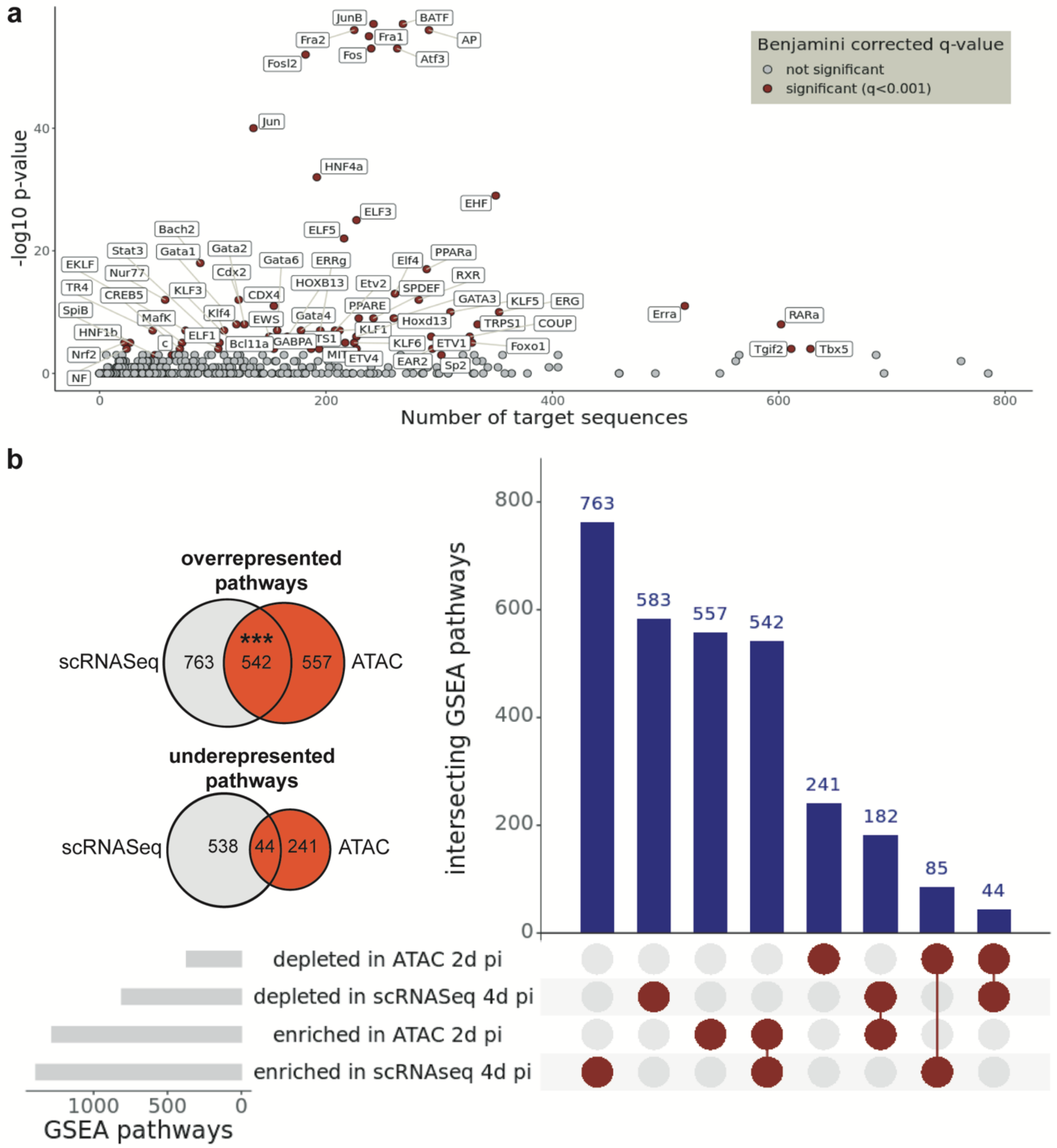

Supplementary Fig. 7. ATAC sequencing of control and N-terminal mutant (D164A) crypts.

a) Results of HOMER motif enrichment on 1469 differential ATAC peaks (logFC > |1| & p < 0.01, as calculated by edgeR[2]'s exact test) between control and N-terminal mutant (D164A) crypts. Number of target sequences on x-axis, -log10(p-value) of enrichment on y-axis. Significantly enriched motifs (Benjamini-corrected q-value < 0.001) depicted in red and labeled with corresponding transcription factors.

b) Venn diagrams and upset plot showing overlaps between enriched pathways (logFC > |1|) in N-terminal mutant (D164A) crypts, compared to controls, as revealed by ATACSeq (timepoint 2d pi) and scRNASeq (timepoint 4d pi). Significance of the overlap of overrepresented pathways calculated with two-sided hypergeometric test (*** p < 2.2e-16).

Supplementary Table 1

REAGENT or RESOURCE	SOURCE	IDENTIFIER
**Antibodies**		
mouse monoclonal anti β-catenin (C-terminus)	BD Transduction Lab	610153
rabbit monoclonal anti β-catenin (N-terminus)	Novus	NBP1-32239
mouse monoclonal anti-lamin A/C	eBioscience	14-9847-82
mouse monoclonal anti-β-actin	Santa Cruz Biotech.	sc-47778
rabbit polyclonal anti Ki67	Abcam	ab15580
mouse monoclonal anti E-cadherin	BD Transduction Lab	610181
rabbit polyclonal anti Lysozyme	Dako	A0099
rat monoclonal anti CD45-FITC	Invitrogen	12-0453-82
rabbit Sox9	Sigma Aldrich	AB5535
rabbit monoclonal anti-Epcam	Abcam	AB213500
rabbit monoclonal anti-Olfm4	Cell Signaling	39141
rabbit polyclonal anti-ACTIVE® JNK pAb	Promega	V7931
Alexa fluor 594 goat anti-rabbit	ThermoFischer	A-11037
Alexa fluor 647 goat anti-mouse	ThermoFischer	A-21236
Alexa fluor 594 goat anti-rat	ThermoFischer	A-11007
Peroxidase-AffiniPure Goat Anti-Rabbit IgG	Jackson ImmunoResearch	111-035-144
**Chemicals**		
Tamoxifen	Sigma	T5648
Matrigel	Corning	354230
4-hydroxytamoxifen	Sigma	H6278
ROCK inhibitor Y-27632	Millipore	SCM075
Alcian Blue	Sigma	A3157
Hematoxylin	Sigma	51275
Eosin Y-solution	Sigma	HT110280
DAPI	Sigma	D9543
4% paraformaldehyde in PBS	Santa Cruz Biotechnology	sc-281692
TripLE Express Enzyme 1X	ThermoFischer	12604013
Advanced DMEM/F12	ThermoFischer	12634010
Tissue-Tek OCT Compound	Sakura	4583
Bovine Serum Albumin	Sigma	A9647
Gentle Cell Dissociation Reagent	Stemcell Technologies	07174
Instesticult Organoid Growth Medium (Mouse)	Stemcell Technologies	06005
**Commercial Assay**		
VECTASTAIN ABC HRP Kit	Vector Laboratories	PK-4000
RNAScope 2.5 HD Reagent Kit Brown	ACDBio	322300
MinElute PCR Purification Kit	Qiagen	28004
WesternBright Quantum HRP substrate	advansta	K-12042
Edu Click 647 Kit + EdU	baseclick GmbH	BCK647-IV-IM
**Deposited Data**		
bulk RNA sequencing	This study	GEO: GSE148941
single cell mRNA sequencing	This study	GEO: GSE148942

bulk ATAC sequencing	This study	GEO: GSE148940
**Experimental Models: Duodenal Organoid Lines**		
Murine VillinCre-ER^{T2}-Ctnnb1$^{D164A/flox}$	This study	N/A
Murine VillinCre-ER^{T2}-Ctnnb1$^{wt/flox}$	This study	N/A
Murine Ctnnb1$^{wt/wt}$	This study	N/A
Murine Ctnnb1$^{wt/KO}$	This study	N/A
Murine Ctnnb1$^{dm/flox}$	This study	N/A
Murine Ctnnb1$^{\Delta C/flox}$	This study	N/A
**Experimental Models: Organisms/Strains**		
Mus musculus_Ctnnb1-D164A	Valenta *et al,* 2011[5]	RRID:MGI: 5308942
Mus musculus_Ctnnb1-delC	Valenta *et al,* 2011[5]	RRID:MGI: 5308945
Mus musculus_Ctnnb1-dm	Valenta *et al,* 2011[5]	RRID:MGI: 5308947
Mus musculus_Ctnnb1-flox	Brault *et al,* 2001[1]	RRID:MGI: 2148567
Mus musculus_Tg(Vil1-cre/ERT2)	The Jackson Laboratory	RRID:MGI: 3053826
Mus musculus_ BCL9-loxP_BCL9L-loxP	Deka *et al,* 2010[6]	RRID:MGI: 4398979
**Genotyping Primers**		
Fwd primer Ctnnb1-D164A: TCCCTGAGACGCTAGATG	Valenta *et al,* 2011[5]	N/A
Rev primer Ctnnb1-D164A: GAGTCCCAGCAGTACAAC	Valenta *et al,* 2011[5]	N/A
Fwd primer Ctnnb1-delC: GTGCACACGTCATGCTTTAC	Valenta *et al,* 2011[5]	N/A
Rev primer Ctnnb1-delC: TGGCTTGTCCTCAGACATTCG	Valenta *et al,* 2011[5]	N/A
Fwd primer vilCreER^{T2}: CAAGCCTGGCTCGACGGCC	El Marjou *et al.*, 2004[7]	N/A
Rev primer vilCreER^{T2}: CGCGAACATCTTCAGGTTCT	El Marjou *et al.*, 2004[7]	N/A
Primer RM41 Ctnnb1-flox: AAGGTAGAGTGATGAAAGTTGTT	Brault *et al*. 2001[1]	N/A
Primer RM42 Ctnnb1-flox: CACCATGTCCTCTGTCTATTC	Brault *et al*. 2001[1]	N/A
Primer RM43 Ctnnb1-flox: TACACTATTGAATCACAGGGACTT	Brault *et al*. 2001[1]	N/A
Fwd primer Bcl9-loxP: CCTGCCGAGATGGTCTCAGTTC	Deka *et al,* 2010[6]	N/A
Rev primer Bcl9-loxP: CACCCCAGGCTACCTCACTGAC	Deka *et al,* 2010[6]	N/A
Fwd primer Bcl9L-loxP: CAACCCACCGGGACCTCTC	Deka *et al,* 2010[6]	N/A
Rev primer Bcl9L-loxP: GGAGGAGCGGAGGAGCTGTTC	Deka *et al,* 2010[6]	N/A
**qRT-PCR primers**		
RT primer Axin2 fwd: GGGGGAAAACACAGCTTACA	Valenta *et al,* 2011[5]	N/A
RT primer Axin2 rev: ACTGGGTCGCTTCTCTTGAA	Valenta *et al,* 2011[5]	N/A
RT primer Lgr5 fwd: CTCCACACTTCGGACTCAACAG	Valenta *et al,* 2011[5]	N/A
RT primer Lgr5 rev: AACCAAGCTAAATGCACCGAAT	Valenta *et al,* 2011[5]	N/A
RT primer Olfm4 fwd: GCCACTTTCCAATTTCAC	Valenta *et al,* 2011[5]	N/A
RT primer Olfm4 rev: GAGCCTCTTCTCATACAC	Valenta *et al,* 2011[5]	N/A
RT primer Ascl2 fwd: TGGCACGCCGCAATG	Valenta *et al,* 2016[8]	N/A
RT primer Ascl2 rev: CCTGGAAGCCCAAGTTTACC	Valenta *et al,* 2016[8]	N/A
RT primer Cd44 fwd: TCCTTCTTTATCCGGAGCAC	Degirmenci *et al,* 2018[9]	N/A
RT primer Cd44 rev: ACGTCTCCTGCTGGGTAGC	Degirmenci *et al,* 2018[9]	N/A
RT primer Hspa5 fwd: GAGGATGTGGGCACGGTGGT	This study	N/A
RT primer Hspa5 rev: CCCTGATCGTTGGCTATGAT	This study	N/A
RT primer Ddit3 fwd: CATACACCACCACACCTGAAAG	This study	N/A
RT primer Ddit3 rev: CCGTTTCCTAGTTCTTCCTTGC	This study	N/A
RT primer GAPDH fwd: AACTTTGGCATTGTGGAAGG	Valenta *et al,* 2011[5]	N/A
RT primer GAPDH rev: ATCCACAGTCTTCTGGGTGG	Valenta *et al,* 2011[5]	N/A

See Supplementary Data 1 for smFISH probes	This study	N/A
**Software and Algorithms**		
GraphPad Prism v7.0a	GraphPad Software Schneider	https://www.graphpad.com/scientific-software/prism/
IGV 2.8.0	Broad Institute	https://software.broadinstitute.org/software/igv/
R software 3.6.1	GNU project	https://www.r-project.org
R Studio	RStudio	https://www.rstudio.com
SUSHI: Supporting User for SHell script Integration	Functional Genomics Center Zurich	https://github.com/uzh/sushi
edgeR R package	Robinson *et al,* 2009[2]	https://bioconductor.org/packages/release/bioc/html/edgeR.html
pheatmap R package	Kolde, 2012[10]	https://cran.r-project.org/web/packages/pheatmap/pheatmap.pdf
Seurat v3.0	Stuart *et al,* 2019[11]	https://satijalab.org/seurat/get_ started.html RRID:SCR_016341
conos R package	Barkas *et al,* 2019[12]	https://github.com/hms-dbmi/conos
psupertime R package	Mcnair & Claassen, 2018[13]	https://github.com/wmacnair/psupertime
destiny R package	Angerer *et al,* 2015[14]	http://bioconductor.org/packages/release/bioc/html/destiny.html
msigdbr R package	R Bioconductor	https://cran.r-project.org/web/packages/msigdbr/vignettes/msigdbr-intro.html
fgsea R package	Sergushichev *et al,* 2016[15]	https://bioconductor.org/packages/release/bioc/html/fgsea.html
ggplot2 R package	Wickham, 2016[16]	https://cloud.r-project.org/web/packages/ggplot2/index.html
bedtools	Quinlan and Hall, 2010[17]	https://bedtools.readthedocs.io/en/latest/
HOMER v4.11	Brenner *et al,* 2017[18]	http://homer.ucsd.edu/homer/
LAS-X	Leica Microsystems	https://www.leica-microsystems.com/products/microscope-software/
Image J Fiji	Schindelin *et al,* 2012[19]	https://imagej.net/Fiji/
inForm Cell Analysis	Perkin Elmer	https://www.perkinelmer.com/lab-solutions/resources/docs/BRO_010576_01_PRD_inForm.pdf

Supplementary References

1. Brault, V. *et al.* Inactivation of the beta-catenin gene by Wnt1-Cre-mediated deletion results in dramatic brain malformation and failure of craniofacial development. *Development* **128**, 1253–1264 (2001).
2. Robinson, M. D., McCarthy, D. J. & Smyth, G. K. edgeR: A Bioconductor package for differential expression analysis of digital gene expression data. *Bioinformatics* (2009) doi:10.1093/bioinformatics/btp616.
3. Chen, E. Y. *et al.* Enrichr: Interactive and collaborative HTML5 gene list enrichment analysis tool. *BMC Bioinformatics* (2013) doi:10.1186/1471-2105-14-128.
4. Kuleshov, M. V. *et al.* Enrichr: a comprehensive gene set enrichment analysis web server 2016 update. *Nucleic Acids Res.* (2016) doi:10.1093/nar/gkw377.
5. Valenta, T. *et al.* Probing transcription-specific outputs of β-catenin in vivo SUPPLEMENTARY. *Genes Dev.* **1**, (2011).
6. Deka, J. *et al.* Bcl9/Bcl9l are critical for Wnt-mediated regulation of stem cell traits in colon epithelium and adenocarcinomas. *Cancer Res.* **70**, 6619–6628 (2010).
7. El Marjou, F. *et al.* Tissue-specific and inducible Cre-mediated recombination in the gut epithelium. *Genesis* **39**, 186–193 (2004).
8. Valenta, T. *et al.* Wnt Ligands Secreted by Subepithelial Mesenchymal Cells Are Essential for the Survival of Intestinal Stem Cells and Gut Homeostasis. *Cell Rep.* **15**, 911–918 (2016).
9. Degirmenci, B., Valenta, T., Dimitrieva, S., Hausmann, G. & Basler, K. GLI1-expressing mesenchymal cells form the essential Wnt-secreting niche for colon stem cells. *Nature* **558**, 449–453 (2018).
10. Kolde, R. Package `pheatmap’. *Bioconductor* (2012).
11. Stuart, T. *et al.* Comprehensive Integration of Single-Cell Data. *Cell* (2019) doi:10.1016/j.cell.2019.05.031.
12. Barkas, N. *et al.* Joint analysis of heterogeneous single-cell RNA-seq dataset collections. *Nat. Methods* (2019) doi:10.1038/s41592-019-0466-z.
13. Macnair, W. & Claassen, M. psupertime: supervised pseudotime inference for single cell RNA-seq data with sequential labels. *bioRxiv* (2019) doi:10.1101/622001.
14. Angerer, P. *et al.* Destiny: Diffusion maps for large-scale single-cell data in R. *Bioinformatics* (2016) doi:10.1093/bioinformatics/btv715.
15. Sergushichev, A. A. An algorithm for fast preranked gene set enrichment analysis using cumulative statistic calculation. *bioRxiv* (2016) doi:10.1101/060012.
16. Wickham, H. *ggplot2: elegant graphics for data analysis*. *Journal of the Royal Statistical Society: Series A (Statistics in Society)* (2016). doi:10.1007/978-3-319-24277-4.
17. Quinlan, A. R. & Hall, I. M. BEDTools: A flexible suite of utilities for comparing genomic features. *Bioinformatics* (2010) doi:10.1093/bioinformatics/btq033.
18. Benner, C., Heinz, S. & Glass, C. K. HOMER - Software for motif discovery and next generation sequencing analysis. *Http://Homer.Ucsd.Edu/* (2017).
19. Schindelin, J. *et al.* Fiji: An open-source platform for biological-image analysis. *Nature Methods* (2012) doi:10.1038/nmeth.2019.
