# Supplementary material for: Differential regulation of β-catenin-mediated transcription via N- and C-terminal co-factors governs identity of murine intestinal epithelial stem cells
Source: Nat Commun. 2021 Mar 1;12:1368. doi: 10.1038/s41467-021-21591-9 (PMC7921392; doi:10.1038/s41467-021-21591-9)
Supplement: Supplementary file 3 — Reporting Summary [file 41467_2021_21591_MOESM3_ESM.pdf]

nature research

Corresponding author(s): Tomas Valenta, Konrad Basler

Last updated by author(s): Jan 28, 2021

Reporting Summary

Nature Research wishes to improve the reproducibility of the work that we publish. This form provides structure for consistency and transparency in reporting. For further information on Nature Research policies, see our Editorial Policies and the Editorial Policy Checklist.

Statistics

For all statistical analyses, confirm that the following items are present in the figure legend, table legend, main text, or Methods section.

n/a	Confirmed	
☐	☒	The exact sample size (*n*) for each experimental group/condition, given as a discrete number and unit of measurement
☐	☒	A statement on whether measurements were taken from distinct samples or whether the same sample was measured repeatedly
☐	☒	The statistical test(s) used AND whether they are one- or two-sided<br>*Only common tests should be described solely by name; describe more complex techniques in the Methods section.*
☒	☐	A description of all covariates tested
☐	☒	A description of any assumptions or corrections, such as tests of normality and adjustment for multiple comparisons
☐	☒	A full description of the statistical parameters including central tendency (e.g. means) or other basic estimates (e.g. regression coefficient) AND variation (e.g. standard deviation) or associated estimates of uncertainty (e.g. confidence intervals)
☐	☒	For null hypothesis testing, the test statistic (e.g. *F*, *t*, *r*) with confidence intervals, effect sizes, degrees of freedom and *P* value noted<br>*Give P values as exact values whenever suitable.*
☒	☐	For Bayesian analysis, information on the choice of priors and Markov chain Monte Carlo settings
☒	☐	For hierarchical and complex designs, identification of the appropriate level for tests and full reporting of outcomes
☒	☐	Estimates of effect sizes (e.g. Cohen's *d*, Pearson's *r*), indicating how they were calculated

Our web collection on statistics for biologists contains articles on many of the points above.

Software and code

Policy information about availability of computer code

Data collection	No software was used.
Data analysis	The code used in this study is available at the public repository https://github.com/cocoborrelli/betacat (doi: 10.5281/zenodo.4461476).<br><br>Sofwares:<br>GraphPad Prism v7.0a and v8.4,3 GraphPad Software Schneider https://www.graphpad.com/scientific- software/prism/<br>IGV 2.8.0 Broad Institute https://software.broadinstitute.org/software/igv/<br>R software 3.6.1 GNU project https://www.r-project.org<br>R Studio RStudio Version 1.1.463 https://www.rstudio.com<br>FastQC, RSEMApp and edgeRApp within SUSHI: Supporting User for SHell script Integration Functional Genomics Center Zurich v2.0.0 https://github.com/uzh/sushi<br>EnrichR Chen et al, 2013, Kuleshov et al, 2016 https://maayanlab.cloud/Enrichr/<br>edgeR R package Robinson et al, 2010 https://bioconductor.org/packages/release/bioc/html/edgeR.html<br>pheatmap R package Kolde, 2012 https://cran.r-project.org/web/packages/pheatmap/pheatmap.pdf<br>Seurat v3.0 Stuart et al, 2019 https://satijalab.org/seurat/get_ started.html RRID:SCR_016341<br>conos R package Barkas et al, 2019 https://github.com/hms-dbmi/conos<br>psupertime R package Mcnair & Claassen, 2018 https://github.com/wmacnair/psupertime<br>destiny R package Angerer et al, 2015 http://bioconductor.org/packages/release/bioc/html/destiny.html<br>msigdbr R package R Bioconductor https://cran.r-project.org/web/packages/msigdbr/vignettes/msigdbr-intro.html<br>fgsea R package Sergushichev et al, 2016 https://bioconductor.org/packages/release/bioc/html/fgsea.html<br>ggplot2 R package Wickham, 2016 https://cloud.r-project.org/web/packages/ggplot2/index.html<br>bedtools Quinlan and Hall, 2010 https://bedtools.readthedocs.io/en/latest/

HOMER v4.11 Heinz et al, 2010 http://homer.ucsd.edu/homer/
LAS-X Leica Microsystems https://www.leica-microsystems.com/products/microscope-software/
Image J Fiji Schindelin et al, 2012 https://imagej.net/Fiji/
inForm Cell Analysis Perkin Elmer https://www.perkinelmer.com/lab-solutions/resources/docs/BRO_010576_01_PRD_inForm.pdf
FlowJo v10.6.2 (Becton Dickinson & Company)

For manuscripts utilizing custom algorithms or software that are central to the research but not yet described in published literature, software must be made available to editors and reviewers. We strongly encourage code deposition in a community repository (e.g. GitHub). See the Nature Research guidelines for submitting code & software for further information.

Data

Policy information about availability of data

All manuscripts must include a data availability statement. This statement should provide the following information, where applicable:

- Accession codes, unique identifiers, or web links for publicly available datasets
- A list of figures that have associated raw data
- A description of any restrictions on data availability

Data generated in this study are deposited in GEO with the accession numbers:
- bulkRNASeq: GSE148941 https://www.ncbi.nlm.nih.gov/geo/query/acc.cgi?acc=GSE148941
- single cell RNASeq: GSE148942 https://www.ncbi.nlm.nih.gov/geo/query/acc.cgi?acc=GSE148942
- ATACSeq: GSE148940 https://www.ncbi.nlm.nih.gov/geo/query/acc.cgi?acc=GSE148940

The remaining data are available within the Article, Supplementary Information or available from the authors upon request. Source data are provided with this paper. Source data are provided in the Source Data File.

For GSEA, gene sets within Molecular Signature Database were used (Liberzon, A. et al. The Molecular Signatures Database Hallmark Gene Set Collection. Cell Syst. (2015) doi:10.1016/j.cels.2015.12.004.)

Field-specific reporting

Please select the one below that is the best fit for your research. If you are not sure, read the appropriate sections before making your selection.

[x] Life sciences [ ] Behavioural & social sciences [ ] Ecological, evolutionary & environmental sciences

For a reference copy of the document with all sections, see nature.com/documents/nr-reporting-summary-flat.pdf

Life sciences study design

All studies must disclose on these points even when the disclosure is negative.

Sample size	In accordance with the 3Rs, the smallest sample size was chosen that could give a significant difference. Given the robustness of the phenotypes across all methods used (on chromatin, transcriptome and protein level), the minimum sample size assuming no overlap in control versus experimental is three animals per experiment.
Data exclusions	Animals were excluded if recombination of the conditional floxed beta-catenin allele was not confirmed by PCR post mortem (pre-established criterium). This resulted in experimental groups with only 2 animals for experiments shown in Fig 1d, 4e-g, Supplementary Fig. 6b. In these cases, independent validation on additional animals was carried out, for example by QRT-PCR.
Replication	Data was combined from at least 3 independent mice, induced on different days and analyzed together. All attempts of replication were successful.
Randomization	Randomly chosen animals of particular genotype were used. All animals were age (8-12 weeks) and sex matched (both males and females were used).
Blinding	The researcher was blinded to the genotype during the processing and analysis.

Reporting for specific materials, systems and methods

We require information from authors about some types of materials, experimental systems and methods used in many studies. Here, indicate whether each material, system or method listed is relevant to your study. If you are not sure if a list item applies to your research, read the appropriate section before selecting a response.

Materials & experimental systems

n/a	Involved in the study	
	☒	Antibodies
☒		Eukaryotic cell lines
☒		Palaeontology and archaeology
	☒	Animals and other organisms
☒		Human research participants
☒		Clinical data
☒		Dual use research of concern

Methods

n/a	Involved in the study	
☒		ChIP-seq
	☒	Flow cytometry
☒		MRI-based neuroimaging

Antibodies

Antibodies used

Primary antibodies for immunohistochemistry (1:100) and immunoblotting (1:1000):
mouse monoclonal anti β-catenin (C-terminus, clone 14) BD Transduction Lab 610153
rabbit polyclonal anti β-catenin (N-terminus) Novus NBP1-32239
mouse monoclonal anti-lamin A/C (3A6-4C11) eBioscience 14-9847-82
mouse monoclonal anti-β-actin (C4) Santa Cruz Biotech. sc-47778
rabbit polyclonal anti Ki67 Abcam ab15580
mouse monoclonal anti E-cadherin (clone 36) BD Transduction Lab 610181
rabbit polyclonal anti Lysozyme Dako A0099
rat monoclonal anti CD45.1 (A20) FITC Invitrogen 12-0453-82
rabbit polyclonal Sox9 Sigma Aldrich AB5535
rabbit monoclonal anti-Epcam (EPR20532-222) Abcam AB213500
rabbit monoclonal anti-Olfm4 (D6Y5A) Cell Signaling 39141
rabbit polyclonal anti-ACTIVE® JNK pAb Promega V7931

Secondary antibodies:
Alexa fluor 594 goat anti-rabbit ThermoFischer A-11037
Alexa fluor 647 goat anti-mouse ThermoFischer A-21236
Alexa fluor 594 goat anti-rat ThermoFischer A-11007
Peroxidase-AffiniPure Goat Anti-Rabbit IgG Jackson ImmunoResearch 111-035-144

Antibodies used for FACS were all from BioLegend (1:200):
anti-mouse CD16/32 (clone 93, 101302) B220 (RA3-6B2, 103246, lot B39 2975), CD11b (M1/70, 101263, lot B29 3812), CD11c (N418, 117333, lot B275102), CD4 (RM4-5, 116012, B273144), CD45 (30-F11, 103151, B293473), F4/80 (BM8, 123116, lot B268075), Ly6G (1A8, 127616, lot B248844), Ly6C (HK1.4, 128018, lot B247616), CD103 (2E7, 121406, B282445), CD8 (53–6.7, 100761, lot B278295), MHC-II (M5/114.15.2, 107622, lot B303311), TCR-β (H57-597, 109222, B281521), IL-17A (TC11-18H10.1, 506916, lot B290261), IFN-γ (XMG1.2, 505830, lot B268951) and TNF-α (MP6-XT22, 506304, lot B271488). Live dead: Fixable Viability Dye eFluor 780 (1:1000, eBioscience, 65-0865-14).

Validation

All antibodies have been previously validated by the manufacturer. Olfm4, Ki67, E-cadherin, β-catenin, Lysozyme, Sox9 anitibodies were validated within our previous work (doi: 10.1038/s41586-018-0190-3 and doi: 10.1016/j.celrep.2016.03.088.)
Our genetic models served as positive/negative controls for some of the stains. The stainings were further supported by qRT-PCR, single cell and bulk RNASeq data, and from chromatin data.

Animals and other organisms

Policy information about studies involving animals; ARRIVE guidelines recommended for reporting animal research

Laboratory animals

Male and female mice >20g mice were induced with tamoxifen from 8 to 12 weeks of age. The following transgenic lines (C57Bl/&J background) were used:
Mus musculus_Ctnnb1-D164A, Valenta et al, 2011
Mus musculus_Ctnnb1-delC, Valenta et al, 2011
Mus musculus_Ctnnb1-dm, Valenta et al, 2011
Mus musculus_Ctnnb1-flox, Brault et al, 2001
Mus musculus_Tg(Vil1-cre/ERT2), The Jackson Laboratory
Mus musculus_ BCL9-loxP_BCL9L-loxP, Deka et al, 2010

Mice were housed in individually ventilated cages, under the standard room temperature (20°-25°C) and humidity, 12/12 light/ dark cycle.

Wild animals

No wild animals were used in this study.

Field-collected samples

No field-collected samples were used in this study.

Ethics oversight

Mouse experiments were performed in accordance with Swiss guidelines and approved by the Veterinarian Office of the Kanton of

Zurich, Switzerland.

Note that full information on the approval of the study protocol must also be provided in the manuscript.

Flow Cytometry

Plots

Confirm that:

- [x] The axis labels state the marker and fluorochrome used (e.g. CD4-FITC).
- [x] The axis scales are clearly visible. Include numbers along axes only for bottom left plot of group (a 'group' is an analysis of identical markers).
- [x] All plots are contour plots with outliers or pseudocolor plots.
- [x] A numerical value for number of cells or percentage (with statistics) is provided.

Methodology

Sample preparation	Control (n=5), ΔC (n=3) and D164A (n=5) mice were sacrificed 2d or 4d pi. The duodenum was open longitudinally, washed and cut into pieces. Peyer's patches were removed. Pieces were weighted and incubated in HBSS with 10% FCS, 100 U/mL penicillin/streptomycin and 5 mM EDTA at 37°C in a shaking incubator. Tissues were then digested at 37°C for 50 min with 15 mM Hepes, an equal mixture of 250 U/mL type IV and type VIII collagenase (Sigma-Aldrich), and 0.05 mg/ml DNase I in RPMI-1640 medium supplemented with 10% FBS and 100 U/ml penicillin/streptomycin. Cells were then layered onto a 40/80% Percoll gradient, centrifuged, and the interface was washed in PBS. Total leukocyte counts were determined by adding countBright Absolute Counting Beads (Life Technologies) to each sample before flow cytometry for normalization to tissue weight. For surface staining, cells were stained in PBS with 0.5% BSA with a fixable viability dye and a combination of the following antibodies: anti-mouse B220 (RA3-6B2), CD11b (M1/70), CD11c (N418), CD4 (RM4-5), CD45 (30-F11), F4/80 (BM8), Ly6G (1A8), Ly6C (HK1.4), CD103 (2E7), CD8 (53–6.7), F4/80 (MB8), MHC-II (M5/114.15.2), TCR-β (H57-597), all from BioLegend. Fc block (anti-CD16/CD32, Affymetrix) was included to minimize nonspecific antibody binding. For intracellular cytokine staining of T cells, cells were incubated for 3.15 h in complete IMDM containing 0.1 µM PMA and 1 µM ionomycin with 1:1,000 brefeldin A (eBioscience) and GolgiStop solutions (BD Biosciences) at 37°C in a humidified incubator with 5% CO2. Following surface staining, cells were fixed and permeabilized with the Cytofix/Cytoperm Fixation/Permeabilization Solution Kit (BD Biosciences) according to the manufacturer's instructions. Cells were then stained for 50 min with antibodies to IL-17A (TC11-18H10.1), IFN-γ (XMG1.2) and TNF-α (MP6-XT22) all from Biolegend.
Instrument	Samples were acquired on a LSRII Fortessa (BD Biosciences)
Software	Aquired data were analyzed using FlowJo software.
Cell population abundance	Absolute numbers of cells are outlined in relevant Figures.
Gating strategy	Events were initially gated by FSC-A and SSC-A, then by FSC-A and FSC-H (to exclude doublets). Live CD45+ cells were then gated using a fixable viability dye. Subsequent gating depends on the population of interest and is outlined in Supplementary Information.

- [x] Tick this box to confirm that a figure exemplifying the gating strategy is provided in the Supplementary Information.
