# Supplementary material for: Differential regulation of β-catenin-mediated transcription via N- and C-terminal co-factors governs identity of murine intestinal epithelial stem cells
Source: Nat Commun. 2021 Mar 1;12:1368. doi: 10.1038/s41467-021-21591-9 (PMC7921392; doi:10.1038/s41467-021-21591-9)
Supplement: Supplementary file 5 — Description of Additional Supplementary Files [file 41467_2021_21591_MOESM5_ESM.pdf]

Title: Supplementary Data 1

Description: This file contains sequences of smFISH probes used for the experiments. For the details of the procedure please see Methods (Single molecule in situ hybridization). Following mRNA were targeted (as indicated in bold): Fos, Junb, Lgr5, Creb3l3
